# Effect of enhanced feedback to hospitals that are part of an emerging clinical information network on uptake of revised childhood pneumonia treatment policy: study protocol for a cluster randomized trial

**DOI:** 10.1186/s13063-017-2152-8

**Published:** 2017-09-07

**Authors:** Philip Ayieko, Grace Irimu, Mike English

**Affiliations:** 1 Kenya Medical Research Institute (KEMRI) – Wellcome Trust Research Programme, Nairobi, Kenya; 2 Department of Paediatrics and Child Health, University of Nairobi, Nairobi, Kenya; 3 Nuffield Department of Medicine, University of Oxford, Oxford, UK

**Keywords:** Audit, Feedback, Pragmatic trial, Cluster trial, Quality of care, Pneumonia, Guidelines

## Abstract

**Background:**

The national pneumonia treatment guidelines in Kenya changed in February 2016 but such guideline changes are often characterized by prolonged delays in affecting practice. We designed an enhanced feedback intervention, delivered within an ongoing clinical network that provides a general form of feedback, aimed at improving and sustaining uptake of the revised pneumonia treatment policy. The objective was to determine whether an enhanced feedback intervention will improve correctness of classification and treatment of childhood pneumonia, compared to an existing approach to feedback, after nationwide treatment policy change and within an existing hospital network.

**Methods/design:**

A pragmatic, cluster randomized trial conducted within a clinical network of 12 Kenyan county referral hospitals providing inpatient pediatric care to children (aged 2–59 months) with acute medical conditions between March and November 2016. The intervention comprised enhanced feedback (monthly written feedback incorporating goal setting, and action planning delivered by a senior clinical coordinator for selected pneumonia indicators) and this was compared to standard feedback (2-monthly written feedback on multiple quality of pediatric care indicators) both delivered within a clinical network promoting clinical leadership linked to mentorship and peer-to-peer support, and improved use of health information on service delivery. The 12 hospitals were randomized to receive either enhanced feedback (*n* = 6) or standard feedback (*n* = 6) delivered over a 9-month period following nationwide pneumonia treatment policy change. The primary outcome is the proportion of all admitted patients with pneumonia (fulfilling criteria for treatment with orally administered amoxicillin) who are correctly classified and treated in the first 24 h. The secondary outcome will be measured over the course of the admission as any change in treatment for pneumonia after the first 24 h.

**Discussion:**

This trial protocol employs a pragmatic trial design during a period of nationwide change in treatment guidelines to address two high-priority areas within implementation research: promoting adoption of health policies and optimizing effectiveness of feedback.

**Trial registration:**

ClinicalTrials.gov, ID: NCT02817971. Registered retrospectively on 27 June 2016

**Electronic supplementary material:**

The online version of this article (doi:10.1186/s13063-017-2152-8) contains supplementary material, which is available to authorized users.

## Background

The slow translation of health research to practice is a major challenge in the provision of quality hospital care in low-income countries [1, 2]. Indeed, experience in Kenya has shown that such health policy changes may take several years to implement [1, 3]. There are several alternative strategies for achieving rapid translation of policy and advancing quality care. Audit and feedback have been well defined and are among the strategies known to impact the adoption of guidance although average effects are modest [4, 5]. The effectiveness of audit and feedback may be influenced by a number of factors including: (i) feedback components, e.g., language, format, and source, (ii) audit components, e.g., data validity, and currency, (iii) nature of behavior change required, and (iv) clearly defined targets, goals and action plans [6]. How best to modify audit and feedback strategies to optimize effectiveness is a priority for research and is of particular relevance as the potential availability of data on multiple aspects of practice within health systems increases.

In collaboration with the Kenyan Ministry of Health and other partners, we have developed an improved system for collecting pediatric inpatient hospital data within a network of Kenyan county hospitals [7]. This clinical information network (CIN) established between 2013 and 2014 has implemented a regular and standardized audit and feedback process comprising performance reports on adherence to guidelines for the care of common conditions causing admission to the hospitals involved. This is linked to additional network activities that together constitute an intervention package aiming to promote adherence to evidence-based practices for all participating hospitals [8, 9]. Working with this network creates opportunities to examine the effects of varying feedback approaches while tracking the adoption of policy [10].

In early 2016, the Ministry of Health in Kenya changed its clinical policy for the treatment of childhood pneumonia. The guideline policy changes reflect new evidence and was agreed by a national clinical guideline panel and involve a reclassification of clinical pneumonia syndromes from the current three grades of severity (very severe pneumonia, severe pneumonia, and pneumonia) to two grades (severe pneumonia equivalent to the previously named very severe grade, and pneumonia, a classification that encompasses the two remaining severity grades) with corresponding changes in recommended antibiotic treatment [11–13]. The new treatment policy recommends orally administered amoxicillin for children who were previously treated with injectable penicillin for pneumonia and who have lower chest wall indrawing but no other serious signs. Using the data collection and semi-automated performance measurement platform provided by CIN, we set out to test specific enhancements of the feedback process including goal setting, benchmarking, and development of clear action plans to examine their effects on the adoption of this change in national clinical guidelines for the treatment of childhood pneumonia [4]. This paper describes a pragmatic, parallel-group, cluster randomized trial to compare the effectiveness of an approach to enhance feedback that might be used in promoting more rapid translation of new treatment policies in routine practice.

### Aims

The overall aim of the trial described in this protocol is to assess the effect of enhancements to feedback on clinician adoption of new pneumonia treatment guidance within county hospitals that are already receiving regular standardized audit reports spanning several clinical conditions. During the study we will examine one primary objective and a secondary objective:

### Primary objective

1. To determine whether a specifically enhanced feedback intervention will improve correctness of classification and treatment of childhood pneumonia compared to standard feedback, after nationwide treatment policy change

### Secondary objective

2. To explore rates of treatment switching after children are started on orally administered amoxicillin for pneumonia as a proxy measure for clinicians’ assessment that treatment failure has occurred on the orally administered treatment

## Methods

**Fig. 1:**
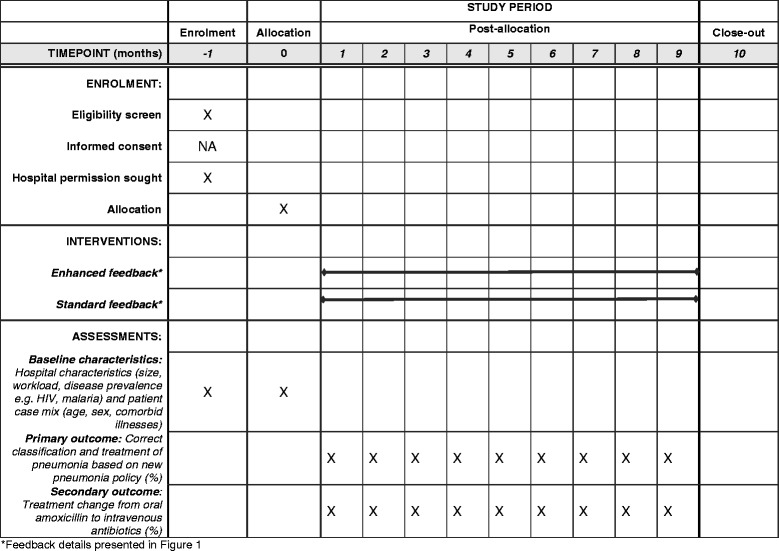
Clinical information network (CIN)-enhanced feedback intervention schedule of enrollment, interventions, and assessments

The reporting of this protocol for the CIN enhanced feedback intervention is conducted according to the Standardized Protocol Items: Recommendations for Intervention Trials (SPIRIT) – Fig. 1 and Additional file 1: Table S1 (SPIRIT 2013 Checklist) [14].

### Study design

**Fig. 2:**
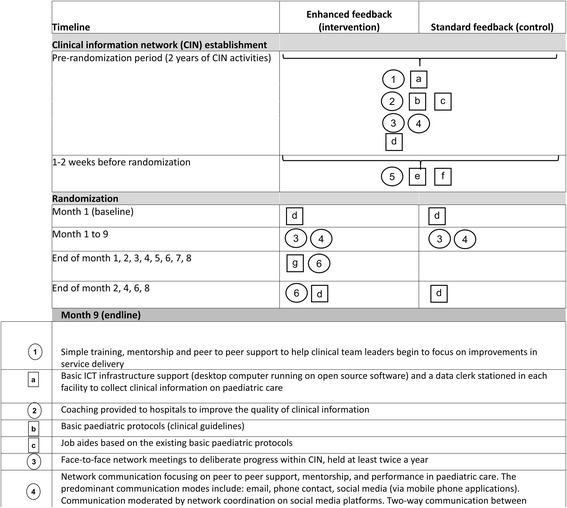
Graphical depiction of interventions in clinical information network (CIN)-enhanced feedback trial (adapted from Perera et al. [25])

We designed a parallel-group, pragmatic, cluster randomized trial to be conducted in 12 hospitals over a 9-month period with interim analysis at 6 months in case of a more marked intervention effect than we are expecting. At the time of the trial design all the 12 hospitals were participating in a clinical network in which standardized audit and feedback had been delivered to all sites for more than 12 months; therefore, providing context for testing an enhanced feedback intervention. The intervention was designed to examine whether specific characteristics of feedback (frequency, goal setting, and action planning) can result in greater impact (Fig. 2). The intervention targets improvements in hospital uptake of new pneumonia treatment policy; therefore, hospitals (and entire pediatric care teams) rather than individual clinicians will be randomized to intervention [15].

### Participants

County referral hospitals form clusters representing diverse geographic regions with varying climatic conditions, morbidity patterns, and facility workload. Out of the 14 hospitals currently engaged in network activities, two hospitals will not participate in the trial because one site located within the capital, Nairobi, serves as a satellite of the main university teaching hospital while the second hospital is a small rural training facility co-located with a larger facility participating in the network. The rural training facility has a different type of patient population and no resident physician. The CIN was set up between February and October 2013 through recruitment of hospitals, based on preliminary consent, from a shortlist of 40 public county referral hospitals providing internship training at the time and serving as first-referral centers. Hospital selection and characteristics are described in more detail elsewhere [16]. The exclusion criteria for hospitals from the eligible population included small hospitals (fewer than 1000 annual pediatric admissions), and existing formal linkages with research or academic institutions working with hospitals to conduct research. The patient population eligible for inclusion within each hospital was pediatric patients admitted with a pneumonia diagnosis in a child with no indication for broad-spectrum antibiotic therapy. Common indications for broad-spectrum antibiotics that will define the exclusion criteria include severe acute malnutrition, and meningitis. Severe malaria and tuberculosis cases will also be excluded. All hospitals recruited in the trial assented to participate and were informed that they could discontinue participation in the trial without prejudicing their role within the CIN.

### Clinical information network intervention and feedback strategies

The CIN comprises a multifaceted approach including audit and feedback for all hospitals to which specifically enhanced feedback on the uptake of new pneumonia treatment policy is added for intervention sites.

#### CIN activities (both study arms)

At the stage of intervention design, two major activities targeting improvement in data collection and clinical management of patients were being implemented (Fig. 2). In summary, each hospital in the network receives, and will continue to receive, five intervention components prior to and during the planned enhanced feedback trial: (1) minimal ICT infrastructure and human resource support, (2) standardized written feedback reports on hospital performance including on adherence to pneumonia guidelines provided every 2 months, (3) national pediatric clinical protocols and job aides based on the protocol, (4) formal network meetings held at least twice a year, and (5) peer-to-peer support through telephone and email contact and networking in social media. A fuller description of these activities and the rationale for employing them is provided elsewhere [9].

#### Network coordination

Since its inception, a senior pediatrician has taken overall responsibility for activities in the network including organizing formal network meetings, providing updates of network activities, disseminating feedback reports, moderating peer-to-peer discussions within network forums (including online communication), providing encouragement, and offering advice as required.

##### Enhanced feedback reporting (intervention arm)

During the entire 9-month trial period we will continue to deliver the network-wide interventions described above including five rounds of general, 2-monthly feedback reports on hospital performance including a baseline feedback report, Fig. 2. Our model of enhancing feedback of hospital performance in the intervention group draws from three notions around effective feedback: clear goal setting (feed up), progress towards goal attainment (feedback), and advising on activities aimed at making better progress (feed forward) [17]. As such, in the intervention arm we will identify specific goals (indicators) aligned with the primary objective, set targets for each focus indicator and benchmark performance for the indicators against a shared target. A specific, additional feedback report on pneumonia policy adherence based on these features will be provided monthly in written format and distributed by email to intervention-hospital pediatricians by the network coordinator together with a follow-up phone call.

The primary target of this intervention is health workers and it will not directly impact on concomitant care and no specific health care intervention will be prohibited as a result of trial participation. At facility level all hospitals will continue receiving the intervention unless assent for participation is withdrawn by the hospital.

#### Adherence to intervention protocol

The strategies that were implemented to improve adherence to intervention protocol include ensuring timeliness of planned audit and feedback cycles, and appropriate training on new policy. A time schedule for delivering written feedback – both standard and enhanced – was prepared during intervention design and automated analysis scripts for producing feedback reports were developed to ensure timeliness in feedback delivery. The Data Management Team will hold weekly meetings during the entire 9-month intervention period to ensure quality of audit data. standard training on the new pneumonia policy will be designed and delivered across the network. A team of pediatricians (GI, SA, AA, and JO) acquainted with development of national pneumonia guidelines who were also qualified in the generic instructor course (GIC) offered by the Resuscitation Council (UK) and Advanced Life Support Group (ALSG) will deliver the training. The CIN coordinator will maintain a communication log documenting feed forward (advise on activities aimed at making better progress).

### Selection of quality performance indicators

The new childhood pneumonia treatment policy advocates for assessment of respiratory rate, lower chest wall indrawing and five specific danger signs (cyanosis, oxygen saturation below 90%, inability to drink/breastfeed, altered level of consciousness, and grunting) in children presenting with cough or difficulty breathing followed by classification into one of two severity classifications: pneumonia (for children with raised respiratory rate and/or chest wall indrawing) or severe pneumonia (for those with any of the remaining danger signs). The guidelines further recommend treatment with orally administered amoxicillin for pneumonia and intravenously administered antibiotics for severe pneumonia [12]. A composite indicator for the classification and treatment of pneumonia was used for the primary outcome. The indicator was developed by reconstructing a pneumonia classification for each patient admitted with cough or difficulty breathing using information documented in medical records on whether each of the six specific clinical signs required for pneumonia severity classification were present or absent (one network effect has much better clinical documentation). This classification, based on a clinical guideline algorithm, was then compared to the classification assigned by the admitting clinician to determine whether the clinician classification was correct. The children with a correct pneumonia classification were then evaluated to determine whether they received orally administered amoxicillin, the recommended treatment for pneumonia, yielding a composite indicator of correct classification and treatment.

### Randomization

The trial will be conducted in 12 geographically dispersed hospitals participating in the CIN. Restricted randomization was used to allocate the participating hospitals into the intervention and control arms to ensure that balance is achieved between the two treatment arms with respect to number of patients admitted as inpatients with pneumonia, and geographical location (also a proxy for malaria prevalence or disease case-mix) [15]. The hospitals were grouped into four categories using information on geographic location (central or western region) and monthly average number of patients admitted with pneumonia (<30 or ≥ 30 per month) collected within the clinical network during the year preceding the intervention. Five hospitals were located in the western region, while seven hospitals had at least 30 patients admitted with pneumonia per month. A statistician used *R* statistical software to generate the 924 possible allocations for assigning 12 clusters to two treatments with six clusters allocated to each treatment arm. We defined a balancing criteria based on the two selected covariates and determined that approximate balance would be achieved by including allocations which had in each treatment arm either two or three hospitals located in the western region and three or four hospitals admitting < 30 pneumonia cases per month. The *R* computer program was used to assess each allocation against the balancing criteria for geographic location (2:3 or 3:2 split between the arms) and monthly number of patients admitted with pneumonia (3:4 or 4:3). There were a total of 536 allocations that met the criteria and represented relative balance between the intervention and control arms with respect to desired allocation ratio. Computer-generated random numbers were used to select one of these allocations at random. Hospitals in the randomly selected allocation were assigned to treatment and those not included in the allocation were assigned to the control group. Allocation was based on clusters rather than individual patients and allocation concealment was conducted at the cluster level. The trial statistician concealed the sequence from investigators until training on the new policy was conducted in all hospitals and the intervention was assigned. Blinding of investigators and health care providers after intervention assignment was not feasible in the trial because the delivery of enhanced feedback on pneumonia care provided was done by investigators and the target of this feedback was the health care providers. However, for the interim analysis the statistician was blinded to treatment arm allocation.

### Sample size

Based on the primary outcome of correct pneumonia classification and treatment we performed a power calculation to determine the feasibility of conducting the trial within the period of the network’s funding cycle using a fixed number of hospitals recruited into CIN. Using data collected during the first year of CIN operation we determined that: on average, 616 (range 310–898) children are admitted to each hospital annually with pneumonia and of these 150 children (range 78–228) are eligible for treatment with amoxicillin. We estimated that if we evaluate the primary outcome 9 months after trial commencement with six hospitals per arm then at least 680 patients with pneumonia requiring amoxicillin treatment will be enrolled per arm. Assuming an average cluster size of 113 patients and an intracluster correlation coefficient (ICC) of 0.2 (corresponding to a variance inflation factor (VIF) of 15.8 and based on calculation of pneumonia classification and treatment within CIN hospitals), a Type-I error rate of 5%, and 90% power we will be able to detect a two-fold increase in the odds of correct pneumonia classification and treatment over 9 months if correct pneumonia classification and treatment is 30% in the control group following the treatment policy change. To ensure that the desired sample size is attained within each hospital, all eligible patients will be recruited in the hospitals with a workload less than 3000 annual pediatric admissions. For hospitals with a very high annual workload (at least 4000 admissions) a random sample will be selected using computer-generated random numbers applied to eligible participants.

### Outcomes

The primary outcome is correct patient assessment and treatment-plan-defining care in the first 24 h. This endpoint was evaluated as the proportion of all patients admitted with pneumonia who were eligible for treatment with orally administered amoxicillin based on guideline recommendations (increased age-specific respiratory rate or lower chest wall indrawing in a child with history of cough or difficulty breathing) who are correctly classified and treated using orally administered amoxicillin at 24 h. The secondary outcome will be measured over the course of the admission as any change in treatment for pneumonia after the first 24 h. This change from orally administered amoxicillin to intravenously administered antibiotics for pneumonia during inpatient stay is considered a proxy for treatment failure rate..

### Data collection, management, and analysis

#### Data collection methods

The trial data were collected at the point of hospital discharge by data clerks using existing data collection tools developed for data collection within the CIN [7, 8]. The clinical tool has been in development and use for some time, and contains information on biodata, clinical features on admission and history of illness [18]. Further data collection procedures were designed for data collection on laboratory investigations, initial antibiotic treatment and supportive care including oxygen therapy for pneumonia, and discharge outcomes after adaptation from previous studies [19]. Prior to the trial commencement the general CIN tools were adapted to capture outcomes to be used for assessing the new pneumonia treatment policy that were not previously contained in the treatment guidelines, e.g., (1) revised pneumonia severity classification and (2) formulation and dosage of dispersible amoxicillin tablets. At the end of each working day (Monday to Friday) a clerk will enter data from inpatient paper records of pediatric discharges into an electronic tool designed using REDCap, a non-proprietary software for clinical data capture. Data for children discharged on weekends will be entered at the start of the new week. All data clerks working in the CIN were retrained on capturing pneumonia data following changes in the pneumonia treatment guidelines. In addition, revised data collection manual and standard operating procedures were available in all sites.

#### Data management

At the end of each day the data clerk at each site synchronized data to a centralized server hosted at the KEMRI-Wellcome Research Program. Data quality was ensured through running error-checking scripts on a daily basis both in the sites and at the centralized trial server and conducting three rounds of external data quality assurance (DQA) during the 9-month trial period. The cleaning scripts raised error queries related to possible out-of-range values and implausible and inconsistent entries. Using the daily error logs generated by the scripts, a data manager flagged the potential errors with the clerk who verified the entries and cleaned any errors. Once all errors had been resolved all data were then archived in the centralized server with the primary data generated at each hospital retained within the hospital. During the periodic external DQA, a team from the KEMRI-Wellcome Trust Research Program visited each site and conducted an independent re-entry of approximately 10% randomly sampled patient data that had been entered by the clerk in the preceding 3 months. The independent entries were then compared to the corresponding existing entries in the database with the aim of determining data quality and identifying database fields where agreement was low for discussion with the data entry clerks.

All hospitals will continue to participate in network activities which will encourage retention of hospitals in the trial. Individual participant retention and follow-up were not major concerns in this trial because the data were collected on discharge through abstracting admission events contained in medical records.

#### Statistical methods

Characteristics of patients and hospitals in the analysis will be compared according to treatment arm to evaluate the ability of randomization to deliver balance across hospital groups. Any variables that show imbalance between intervention groups will be considered for adjustment in comparison of intervention effect.

The primary comparison of correct classification and treatment of pneumonia will include all patients meeting clinical criteria for pneumonia (but not severe pneumonia) recruited in the trial during the 9-month period. The primary outcome will be a binary variable indicating whether a child admitted with non-severe pneumonia is correctly classified and treated based on new guideline recommendations. To account for the cumulative effect of feedback delivered at different time points during the 9-month intervention, the primary comparison will be adjusted for time between commencement of intervention and patient recruitment. A hierarchical logistic regression analysis will be conducted with the primary outcome (correct pneumonia classification and treatment) and independent variables including treatment arm, time (as a continuous variable), and both patient level and hospital level and adjusting for any imbalances in patient characteristics. The model will account for clustering by including a random effect for hospital. The change in performance over time will be included as a time trend. Tests for significant differences in the time trend between the treatment arms will be examined by including an interaction term between the treatment group and the time variable.

In separate sensitivity analysis, multiple imputation will be used to handle missing data and allow classification of all pneumonia patients. Bayesian bootstrap predictive mean matching will be conducted 50 times to impute missing data for signs required to classify pneumonia severity. After imputation, diagnostic checks will be conducted to compare the distribution of imputed and observed data. The modeling approaches used in the primary trial analysis will be repeated after imputation on each imputed dataset and the estimates combined using Rubin’s rules.

#### Monitoring

A formal data monitoring committee was not needed in this trial for the following reasons: the patients meeting the inclusion criteria were non-critical (any danger sign of illness or diagnosis with comorbid illness requiring intravenously administered antibiotics were excluded) and patients were treated for a short time period with amoxicillin, a well-characterized and relatively safe drug known for not having harmful effects in the pediatric age group. Furthermore, the study was on adoption of a national policy recommending amoxicillin use. The primary aim of the interim analysis was to determine whether the minimum sample size required in the study was recruited by 6 months with the possibility of extending the trial for a 3-month period if this was not achieved. This interim analysis was conducted by a statistician blinded to the trial arm allocation.

### Ethical considerations

Scientific and ethical clearance to conduct the trial was obtained from the Scientific and Ethics Review Unit of the Kenya Medical Research Institute. The approval is updated annually and provided for the establishment of the CIN and the conduct of this specific trial within it, and provides for the use of de-identified patient data, obtained through retrospective review of medical records without individual informed consent, for the purposes of service evaluation and implementation research.

## Discussion

The implementation gap that arises from failure to move research findings to practice has been described extensively but such descriptions are rarely followed by deliberate efforts to narrow this gap in low-income settings. This study conducted during a nationwide changeover in childhood pneumonia treatment recommendations is an attempt to explore alternative ways of augmenting the already documented, but modest, effect of feedback on health worker performance in providing care to address this translation problem [4]. Randomized trials within collaborative networks have been conducted in developed countries but, to the best of our knowledge, the current randomized study, implemented in real time during a health policy change, and also based within a pediatric network is novel in low- and middle-income countries [20, 21]. At the global level the findings of this trial should provide timely contributions to the ongoing discourse on how best to optimize the effect of feedback on professional practice and health care outcome [6].

The pragmatic approach to addressing public health questions is increasingly being used because of its ability to demonstrate efficacy of interventions in real-life settings. Specific strengths of the intervention delivered here are the theory-based process for the design of both the network platform and feedback intervention, representation of diverse contexts of the health system through the range of facilities in the trial, and the reduced selection bias associated with randomized allocation to limit selection bias [9, 22–24].

Among the limitations of the trial is the relatively limited number of clusters. This may limit our ability to detect effects and may raise concerns as to the external validity of any findings. It is important to note that most cluster trials in health conducted in low- and middle-income countries to date have involved randomization of communities and hospitals have rarely been used as randomization units. Additionally, there are issues related to the delivery of effective feedback that deserve attention but which this study could not address. These questions include, but are not limited to, the ideal duration of feedback interventions, and alternative suggested key components of feedback that could enhance performance but that were not examined in this trial [4]. In our case, the duration of our intervention was informed by sample size considerations (power, significance level, effect size), and the participating facilities’ pediatric workloads. Inefficiencies may occur if the duration determined under these assumptions is considerably longer than the ideal duration of feedback. Conversely, a short intervention duration might omit the effect of feedback that is lesser in magnitude but still useful at a system level. Providing evidence to address these issues will require continued methodological developments, possibly involving adaptive designs to suit the dynamic nature of feedback interventions.

### Trial status

Ongoing.

## Additional file

Additional file 1: Table S1 SPIRIT 2013 Checklist: recommended items to address in a clinical trial protocol and related documents. (DOC 254 kb)
